# Structure-guided *in vitro* evolution of nanobodies targeting new viral variants

**DOI:** 10.1371/journal.ppat.1012600

**Published:** 2024-09-26

**Authors:** Gang Ye, Fan Bu, Ruangang Pan, Alise Mendoza, Ge Yang, Benjamin Spiller, Brian E. Wadzinski, Lanying Du, Stanley Perlman, Bin Liu, Fang Li

**Affiliations:** 1 Department of Pharmacology, University of Minnesota Medical School, Minneapolis, Minnesota, United States of America; 2 Center for Emerging Viruses, University of Minnesota, Minneapolis, Minnesota, United States of America; 3 Department of Microbiology and Immunology, University of Iowa, Iowa City, Iowa, United States of America; 4 Hormel Institute, University of Minnesota, Austin, Minnesota, United States of America; 5 Department of Pharmacology, Vanderbilt University School of Medicine, Nashville, Tennessee, United States of America; 6 Institute for Biomedical Sciences, Georgia State University, Atlanta, Georgia, United States of America; DUKE-NUS, SINGAPORE

## Abstract

A major challenge in antiviral antibody therapy is keeping up with the rapid evolution of viruses. Our research shows that nanobodies - single-domain antibodies derived from camelids - can be rapidly re-engineered to combat new viral strains through structure-guided *in vitro* evolution. Specifically, for viral mutations occurring at nanobody-binding sites, we introduce randomized amino acid sequences into nanobody residues near these mutations. We then select nanobody variants that effectively bind to the mutated viral target from a phage display library. As a proof of concept, we used this approach to adapt Nanosota-3, a nanobody originally identified to target the receptor-binding domain (RBD) of early Omicron subvariants, making it highly effective against recent Omicron subvariants. Remarkably, this adaptation process can be completed in less than two weeks, allowing drug development to keep pace with viral evolution and provide timely protection to humans.

## Author summary

The ongoing fight against viral diseases is made more difficult by the rapid evolution of viruses, which often outpaces current antiviral therapies. Our study addresses this critical issue by demonstrating the flexibility and rapid adaptability of antiviral nanobodies - single-domain antibodies derived from camelids. We have developed a structure-guided *in vitro* evolution strategy to quickly re-engineer nanobodies, enabling them to neutralize newly emerging viral variants. For viral mutations occurring at nanobody-binding sites, this approach introduces randomized amino acid sequences into nanobody residues near these mutations. Nanobody variants that effectively bind to the mutated viral target are then selected using a phage display library. Using this method, we successfully modified the nanobody Nanosota-3, originally an inhibitor of early Omicron subvariants, into a potent inhibitor of more recent Omicron subvariants. Remarkably, this adaptation process can be completed in under two weeks, significantly narrowing the gap between viral mutation rates and the development of effective therapeutic agents. Our findings have profound implications for antiviral drug development, particularly in light of ongoing global health challenges, providing a pathway to more timely and responsive treatments.

## Introduction

The COVID-19 pandemic has exposed the limitations of conventional antibodies, particularly how viral mutations can swiftly negate the efficacy of existing antibody therapeutics. A striking instance occurred early in the pandemic when several antibody therapeutics effective against earlier variants of SARS-CoV-2 were suddenly rendered obsolete by the emergence of the Omicron variant [1–6]. Antibody treatments target the spike protein on the virus surface, which is crucial for the virus to enter cells by binding to the ACE2 receptor on the cell surface and then fusing the viral and cellular membranes [7–9]. To keep pace with the continuously evolving spike protein, antiviral antibody treatments must either target a universally conserved spike epitope or be swiftly modifiable to address new spike mutations [10–13]. Our research investigates the latter strategy, proposing an innovative method for the rapid adaptation of antibodies to confront newly arising viral mutations.

Nanobodies are single-domain antibodies sourced from camelid animals such as llamas and alpacas [14–16]. Their small size provides multiple advantages over conventional antibodies for antiviral therapy, such as reduced production costs, high thermal stability, and the ability to bind to hidden epitopes on viruses, along with their potential for administration via inhalers [17–19]. Importantly, the simplicity of their single-domain structure makes them ideal for phage display, which is crucial for screening purposes and for *in vitro* evolution [20]. We have recently identified a series of nanobodies that bind to the receptor-binding domain (RBD) of the SARS-CoV-2 spike protein [21,22]. By blocking the interaction between the spike protein and the ACE2 receptor on host cells, these nanobodies effectively neutralize the virus’s ability to enter and infect cells. Notably, one of these nanobodies, named Nanosota-1, was identified from a naive camelid nanobody library using the prototypic SARS-CoV-2 RBD as bait [21]. To enhance its affinity for the RBD, Nanosota-1 underwent two rounds of random *in vitro* maturation, receiving random mutations throughout its coding sequence. However, the indiscriminate nature of these random mutations across the entire nanobody led to an inefficient affinity maturation process. A more rational and targeted approach is essential to adapt nanobodies more effectively to the fast-evolving viral targets.

Nanosota-3 is another SARS-CoV-2 RBD-targeting nanobody that we recently discovered [22]. Derived from an alpaca immunized with the prototypic SARS-CoV-2 spike, Nanosota-3 has demonstrated high potency against both the prototypic SARS-CoV-2 variant and an early Omicron subvariant BA.1, both *in vitro* and in mouse models. However, it is ineffective against a more recent Omicron subvariant XBB.1.5. The cryo-EM structure of prototypic spike in complex with Nanosota-3 revealed the detailed interactions between the two proteins [22]. Therefore, Nanosota-3 is an excellent candidate for structure-guided *in vitro* evolution aimed at countering emerging viral mutations.

In this study, we adapted Nanosota-3 to the XBB.1.5 subvariant using a novel structure-guided *in vitro* evolution approach. We confirmed the potency of the XBB1.5-adapted Nanosota-3 both *in vitro* and in a mouse model, and further elucidated the structural basis for its neutralization of XBB.1.5. This study serves as a proof of concept that structure-guided *in vitro* evolution is a highly effective strategy for equipping nanobodies to match rapid viral evolutions, thereby offering prompt and effective protection for humans.

## Results

### Design and procedure of a novel structure-guided *in vitro* evolution of nanobodies

**Fig 1:**
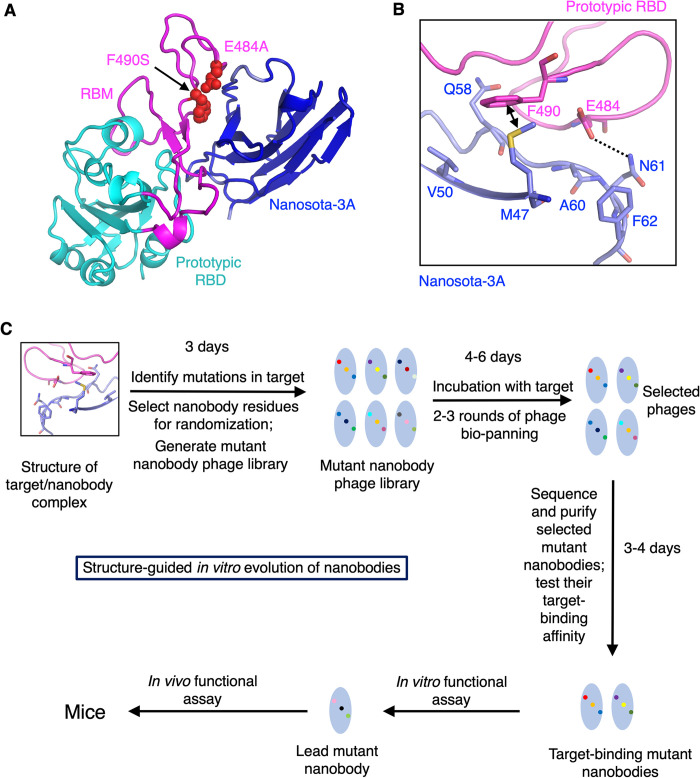
Structure-guided *in vitro* evolution of nanobodies targeting new viral variants. **(A)** Structure of the prototypic SARS-CoV-2 RBD complexed with Nanosota-3A (PDB: 8G73). The core structure and the receptor-binding motif (RBM) of the RBD are shown in cyan and magenta, respectively. Two RBM residues that underwent mutations from prototypic SARS-CoV-2 to Omicron XBB.1.5 within the Nanosota-3-binding epitope are depicted as spheres. **(B)** Six nanobody residues adjacent to the two RBM mutations were selected for *in vitro* evolution. All eight residues are shown as sticks. The dotted line indicates a hydrogen bond, and a double-arrowed line indicates a hydrophobic interaction. **(C)** Flow chart of the structure-guided *in vitro* evolution procedure.

To assess the antiviral spectrum of Nanosota-3, we examined the structural interface between Nanosota-3 and the prototypic SARS-CoV-2 RBD. Compared to the prototypic RBD, XBB.1.5 RBD contains two mutations at this interface: E484A and F490S (Fig 1A). The E484A mutation is likely to disrupt the hydrogen bond formed between Glu484 of the prototypical RBD and Asn61 of Nanosota-3 (Fig 1B). Similarly, the F490S mutation is expected to disrupt the hydrophobic interaction between Phe490 of the prototypical RBD and Met47 of Nanosota-3. Consequently, these two mutations combined significantly reduced Nanosota-3’s affinity for the XBB.1.5 RBD. Therefore, for Nanosota-3 to neutralize XBB.1.5 effectively, it would require engineering to address these specific mutations.

We developed an innovative, structure-based *in vitro* evolution strategy to improve the binding of Nanosota-3 to XBB.1.5 spike (Fig 1C). Our goal was to comprehensively explore all possible combinations of critical amino acid residue changes in Nanosota-3 near the two mutation sites of the XBB.1.5 RBD. We identified six residues on Nanosota-3 for this purpose. In addition to Asn61 and Met47, which directly interact with the mutation sites, we chose four additional residues near the mutation sites on the XBB.1.5 RBD: Ala60 and Phe62 in proximity to the E484A mutation, and Val50 and Gln58 near the F490S mutation. We then constructed a phage display library of Nanosota-3 mutants, which included a fully random assortment of these six residues, and screened for variants that bind with high affinity to XBB.1.5 spike. This screening could be performed using either a single library that includes all six randomized residues or two separate libraries, each randomizing a portion of the six residues. For this study, we opted for the two-library approach, though the single-library method is also feasible.

**Fig 2:**
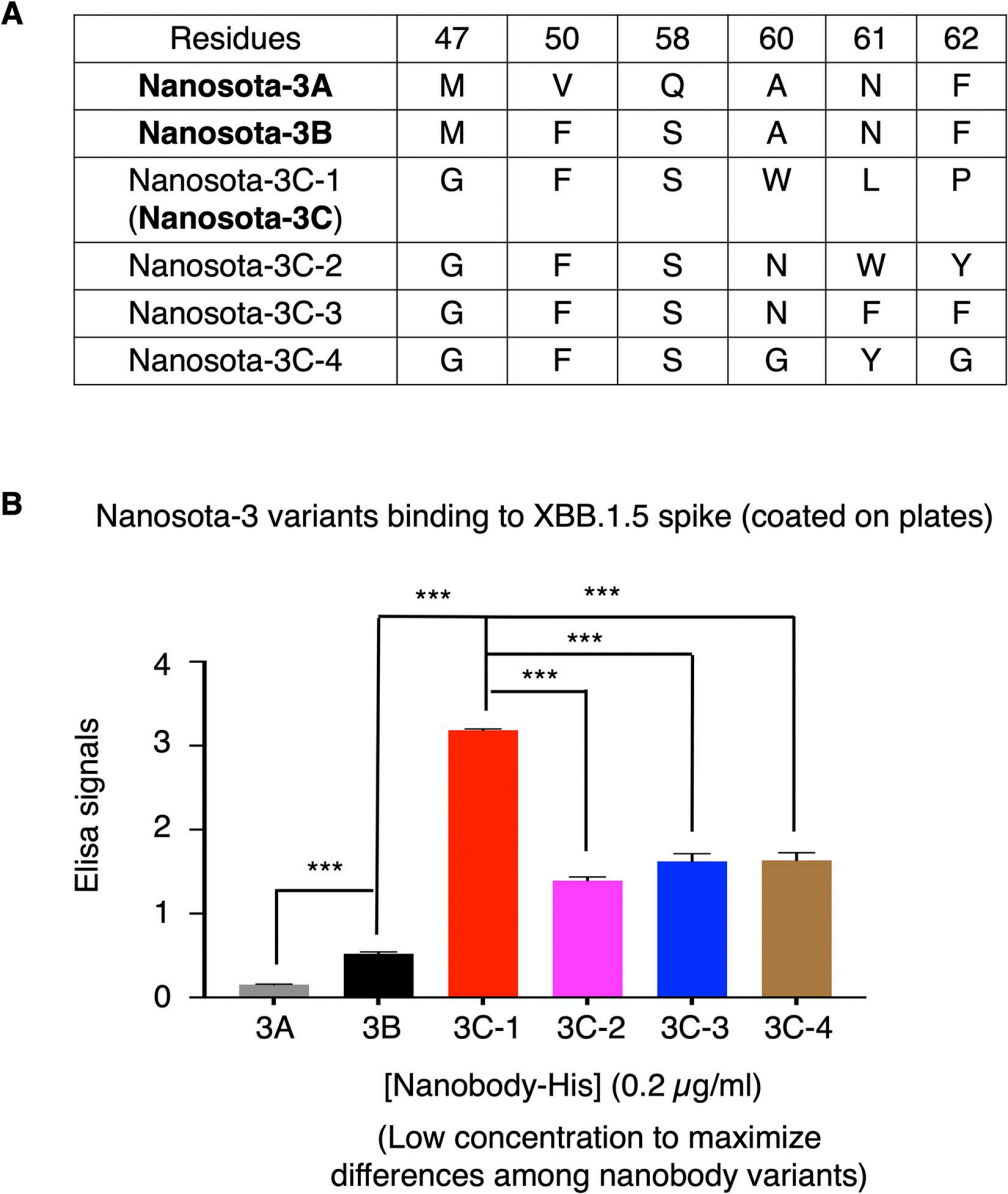
Screening for Nanosota-3 variants that bind to XBB.1.5 spike with high affinity. **(A)** Summary of the mutations in Nanosota-3 selected through the structure-guided *in vitro* evolution process, enabling Nanosota-3 to overcome the two mutations in the XBB.1.5 RBD. **(B)** ELISA between Nanosota-3 variants and the XBB.1.5 spike ectodomain. His-tagged XBB.1.5 spike ectodomain was coated on ELISA plates, followed by the addition of HA- and His-double-tagged Nanosota-3 (Nanosota-3A, -3B, or one of the Nanosota-3C variants). Binding was detected using anti-HA-tag antibodies. Error bars represent SEM (n = 3). A Student’s two-tailed *t*-test was performed to analyze the statistical differences between the indicated groups, with results labeled above each bar. *** *p*< 0.001.

We constructed the first phage display library of nanobody mutants by randomizing two nanobody residues, 50 and 58, surrounding the F490S mutation in the XBB.1.5 RBD. To do this, we introduced mutations into the original Nanosota-3, now referred to as Nanosota-3A, through PCR using primers with fully randomized codons at the intended mutation sites. We then conducted bio-panning of the phages using the XBB.1.5 spike ectodomain as both the target and bait. Phages that showed affinity were sequenced across their nanobody-coding genes, and their respective nanobody variants were produced. These nanobody variants were further assessed through *in vitro* functional assays, including target-binding assays and SARS-CoV-2 pseudovirus entry assays. Among the 96 sequenced target-binding phages, one contained a nanobody with significantly higher binding affinity to the target than Nanosota-3A (Fig 2A and 2B). This top-performing nanobody variant, named Nanosota-3B [22], contains two mutations, V50F and Q58S (Fig 2A), compared to Nanosota-3A. Nanosota-3B was then used as the foundation for the second phage display library, where we randomized four residues, 47, 60, 61, and 62, surrounding the E484A mutation in the XBB.1.5 RBD. After another round of selection, four of the 96 sequenced target-binding phages contained a nanobody that demonstrated significantly improved binding affinity to the target compared to Nanosota-3B (Fig 2A and 2B). The nanobody with the highest binding affinity for XBB.1.5 spike was identified as the top-performing variant, named Nanosota-3C (Fig 2A and 2B). Nanosota-3C contains four mutations - M47G, A60W, N61L, and F62P - compared to Nanosota-3B (Fig 2A).

### Characterization of Nanosota-3C produced through structure-guided *in vitro* evolution

**Fig 3:**
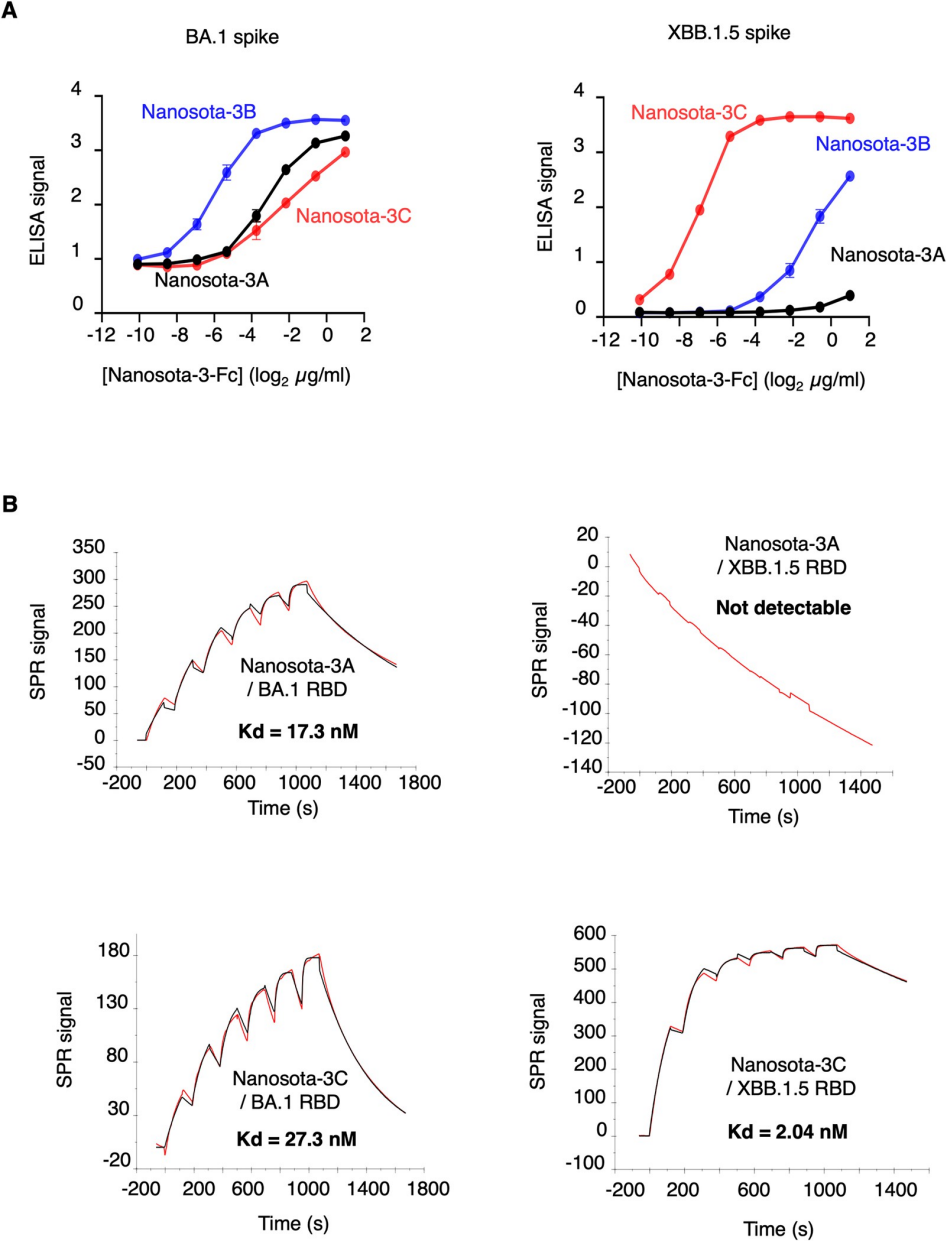
Functional characterization of Nanosota-3’s binding affinity for XBB.1.5 spike. **(A)** Binding interactions between Nanosota-3 variants and Omicron spikes were evaluated using ELISA. His-tagged Omicron spike ectodomain (from the BA.1 or XBB.1.5 subvariant) was coated on ELISA plates, followed by the addition of HA- and His-double-tagged nanobodies (Nanosota-3A, -3B, or -3C). Binding was detected using anti-HA-tag antibodies. Error bars represent SEM (n = 3). **(B)** Binding kinetics between Nanosota-3 variants and Omicron RBDs were determined using surface plasmon resonance (SPR). His-tagged nanobodies (Nanosota-3A or -3C) were immobilized onto a CM5 chip through chemical crosslinking. His-tagged Omicron RBDs (from the BA.1 or XBB.1.5 subvariant) were then injected at various concentrations. The resulting data (red curves) were fitted to a 1:1 binding model (black curves) using Biacore Evaluation Software.

To evaluate the binding affinity of Nanosota-3C with XBB.1.5 spike, we conducted two separate assays. First, we performed an ELISA to assess the binding interactions between Nanosota-3C and both the BA.1 and XBB.1.5 spikes, using Nanosota-3A and -3B as comparisons (Fig 3A). The results showed that all three Nanosota-3 variants bind to BA.1 spike with significant affinity. However, Nanosota-3A, -3B, and -3C bind to XBB.1.5 spike with low, intermediate, and high affinity, respectively. Second, we used surface plasmon resonance (SPR) to quantify the binding interactions between Nanosota-3C and the BA.1 and XBB.1.5 RBDs, with Nanosota-3A serving as a comparison (Fig 3B). The results showed that Nanosota-3C binds strongly to both the BA.1 and XBB.1.5 RBDs, with Kds of 27.3 nM and 2.04 nM, respectively. In comparison, Nanosota-3A binds strongly to the BA.1 RBD but does not bind to the XBB.1.5 RBD. Thus, compared to Nanosota-3A, Nanosota-3C demonstrated dramatically enhanced binding affinity for XBB.1.5 spike.

**Fig 4:**
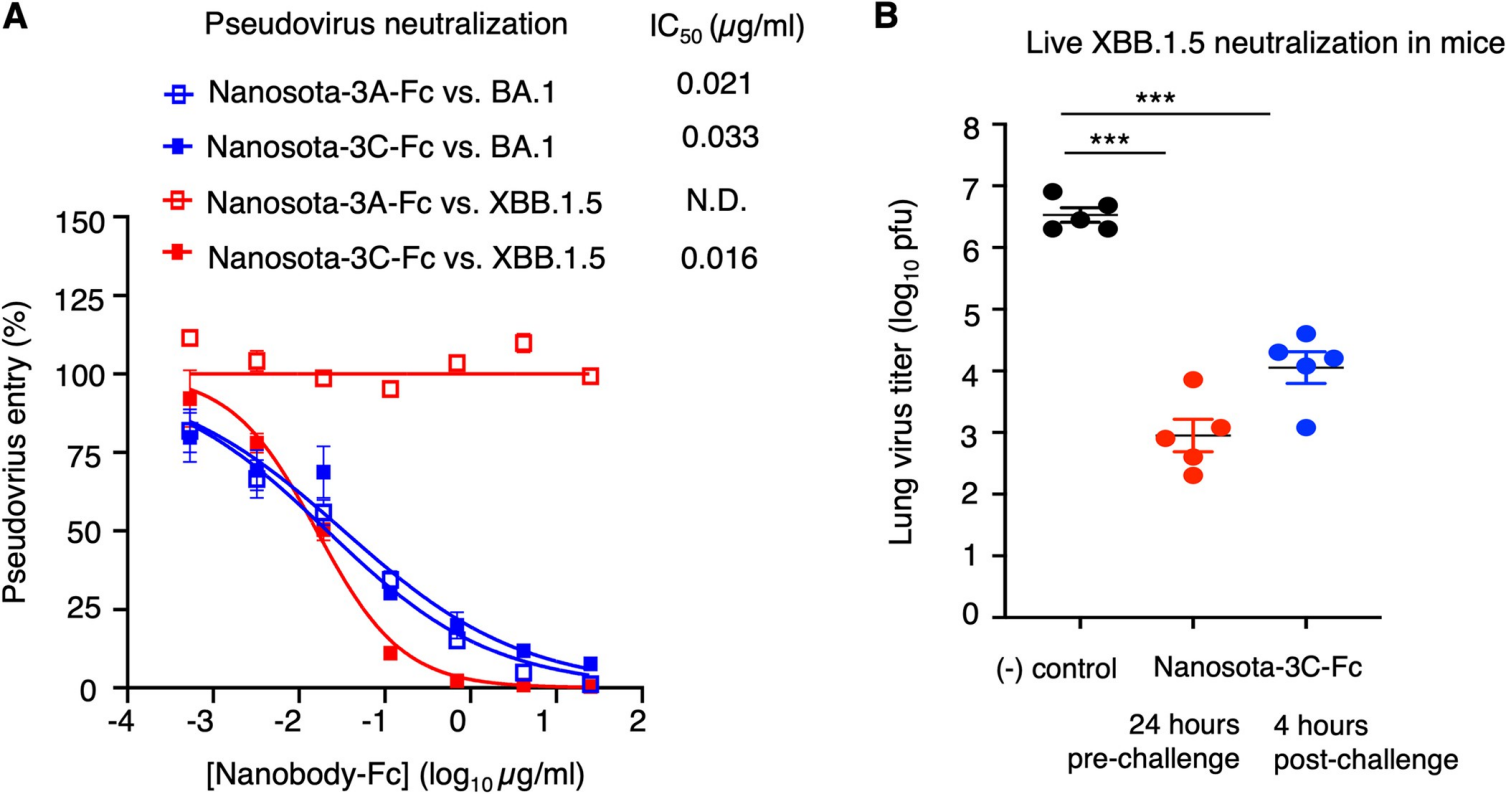
Functional characterization of Nanosota-3’s neutralizing capability against XBB.1.5 entry. **(A)** Efficacy of Nanosota-3A and -3C in neutralizing Omicron pseudoviruses. Retroviruses pseudotyped with Omicron spike (from either the BA.1 or XBB.1.5 subvariant) were used to transduce human ACE2-expressing cells in the presence of Nanosota-3A-Fc or -3C-Fc at various concentrations. Entry efficiency was measured by the accompanying luciferase signal. The efficacy of Nanosota-3A-Fc or -3C-Fc against each pseudovirus type was expressed as the concentration capable of neutralizing pseudovirus entry by 50% (i.e., IC_50_). Error bars represent SEM (n = 3). Each experiment was repeated at least three times, with similar results obtained. **(B)** Efficacy of Nanosota-3C in neutralizing live XBB.1.5 virus in a mouse model. Nanosota-3C-Fc was administered at a dosage of 10 mg/kg body weight either 24 hours pre-challenge or 4 hours post-challenge. C57BL/6 mice were challenged via intranasal inoculation with XBB.1.5 virus. In the treatment group (n = 5), mice received Nanosota-3C-Fc via intraperitoneal delivery. In the control group (n = 5), mice were administered PBS buffer. Lung virus titers on day 2 post-challenge were measured. Comparisons of lung virus titers between the control and treatment groups were performed using an unpaired two-tailed Student’s *t*-test. Error bars represent SEM (n = 5). *** *p*< 0.001.

To evaluate the potency of Nanosota-3C in neutralizing XBB.1.5 entry, we conducted two additional assays. For these tests, Nanosota-3C was modified to include a C-terminal Fc tag, creating Nanosota-3C-Fc. The addition of the Fc tag significantly enhances the *in vivo* half-life of nanobodies by increasing their molecular weight beyond the kidney clearance threshold (~60 kDa) [21]. Despite this increase in size, Fc-tagged dimeric nanobodies remain about half the size of conventional antibodies, and their single-domain antigen-binding sites still effectively access cryptic epitopes on targets. The first assay measured the neutralizing capacity of Nanosota-3C-Fc against XBB.1.5 pseudoviruses (Fig 4A). This involved using retroviruses pseudotyped with XBB.1.5 spike to enter ACE2-expressing cells in the presence of Nanosota-3C-Fc. The results showed that Nanosota-3C-Fc effectively neutralized both BA.1 and XBB.1.5 pseudoviruses, with IC_50_ values of 33 ng/ml and 16 ng/ml, respectively. The second assay assessed the inhibitory potency of Nanosota-3C-Fc against a live XBB.1.5 challenge in a mouse model (Fig 4B). In this experiment, mice were given Nanosota-3C-Fc either 24 hours before or 4 hours after the XBB.1.5 challenge. Analysis of virus titers in their lung tissues 2 days after the challenge revealed that Nanosota-3C-Fc significantly reduced viral titers at both pre-challenge and post-challenge time points. It is important to note that because Omicron variants cause mild symptoms in mice, virus titers in lung tissues were the only metric available for evaluating Omicron infections. Our results suggest that Nanosota-3C is an effective inhibitor of live XBB.1.5 in mice and can be used both as a preventive measure and as a treatment. In summary, Nanosota-3C has demonstrated strong neutralizing effects against the XBB.1.5 subvariant.

### Structural basis of Nanosota-3C’s potent inhibition of XBB.1.5

**Fig 5:**
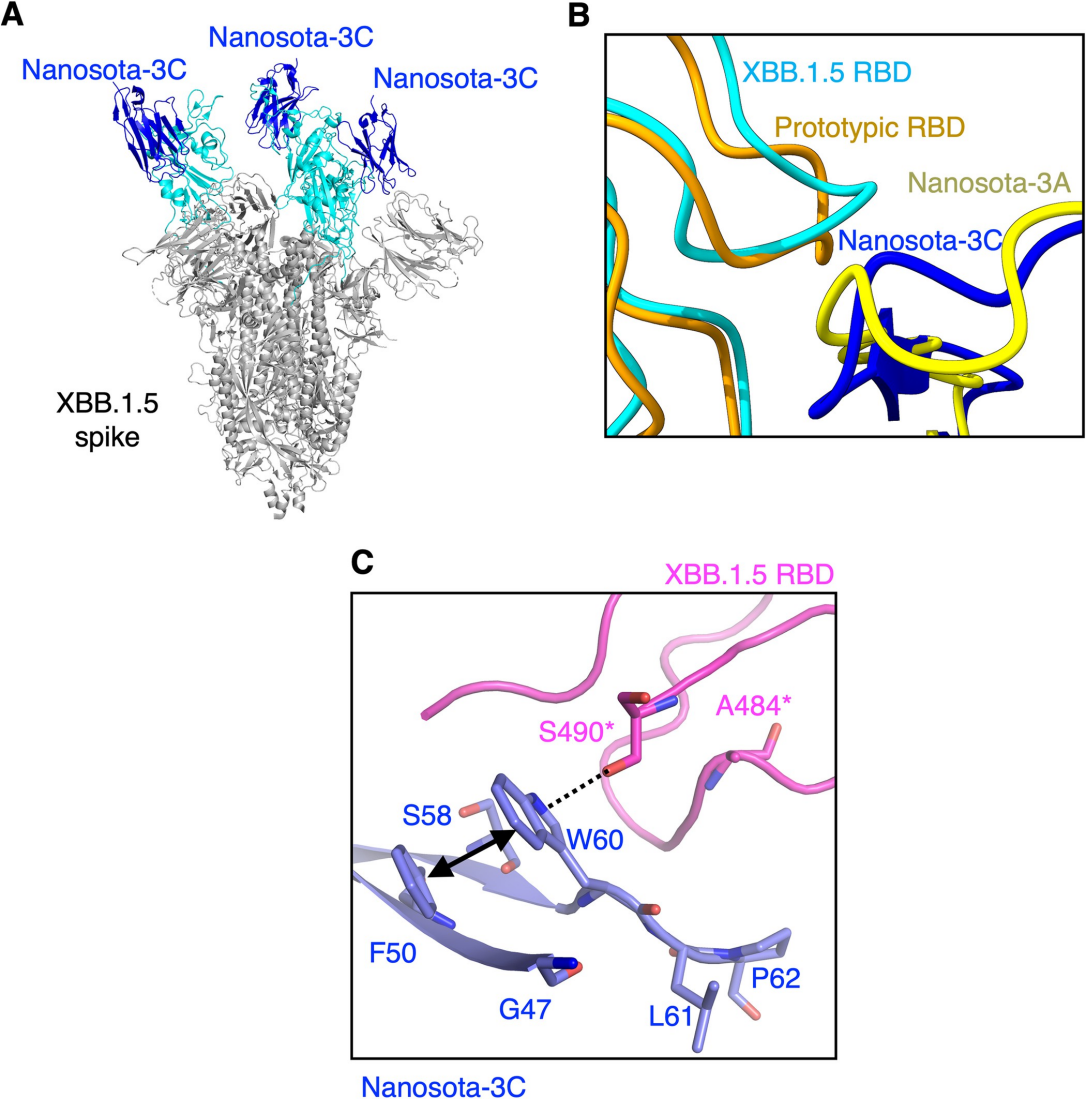
Structure of XBB.1.5 spike complexed with Nanosota-3C. **(A)** The cryo-EM structure of the XBB.1.5 spike ectodomain complexed with Nanosota-3C was determined. XBB.1.5 spike is shown in gray, with the XBB.1.5 RBD in cyan. Among the three copies of the XBB.1.5 RBD, two are in the standing-up conformation, and the third is in the lying-down conformation. Nanosota-3C binds to all three copies of the RBD. **(B)** Superposition of the structures of the prototypic RBD/Nanosota-3A complex and the XBB.1.5 RBD/Nanosota-3C complex in the loop regions where mutations occurred in the XBB.1.5 RBD and Nanosota-3C. **(C)** Detailed structure of the XBB.1.5 RBD/Nanosota-3C interface. Mutations in the XBB.1.5 RBD and Nanosota-3C are shown as sticks. The dotted line indicates a hydrogen bond, and the double-arrowed line indicates a hydrophobic interaction. *To clarify the representation in panel C, we have maintained the labeling of residues 484 and 490 from the prototypic RBD, which correspond to residues 480 and 486 in the XBB.1.5 RBD, respectively. This labeling choice was made to avoid confusion and ensure consistency in our comparisons.

To understand the structural basis for Nanosota-3C’s potent inhibition of XBB.1.5, we determined the cryo-EM structure of the XBB.1.5 spike ectodomain in complex with Nanosota-3C at 3.19Å resolution (Figs 5A and S1 and S1 Table). Our previous research had shown that the RBD of the SARS-CoV-2 spike adopts two conformations: a standing-up position to facilitate receptor binding and a lying-down position to evade the immune system [23,24]. Nanosota-3C, like Nanosota-3A, binds to the RBD in both conformations (Figs 5A and S1). The density at the interface between XBB.1.5 spike and Nanosota-3C is well-defined in the structure (S2 Fig). Compared to the structure of prototypic spike with Nanosota-3A, both XBB.1.5 spike and Nanosota-3C showed significant conformational shifts in the loop regions where mutations are present (Fig 5B). Detailed examination of the interfaces revealed substantial structural changes due to these mutations. In the interface between the prototypic RBD and Nanosota-3A, the interaction was stabilized by hydrophobic contact between Phe490 of the RBD and Met47 of the nanobody, along with a hydrogen bond between Glu484 of the RBD and Asn61 of the nanobody (Fig 1B). However, in the interface between the XBB.1.5 RBD and Nanosota-3C, these interactions were replaced by a π-hydroxyl hydrogen bond between Trp60 of the nanobody and Ser490 of the RBD, and an aromatic π-π stacking interaction between Trp60 and Phe50 of the nanobody (Fig 5C). Thus, the mutations in Nanosota-3C align precisely with the mutations in the XBB.1.5 RBD, enabling high-affinity binding between Nanosota-3C and the XBB.1.5 RBD.

The detailed structural information on the interactions between Nanosota-3C and the XBB.1.5 RBD allowed us to further investigate how each of the *in vitro* evolved mutations contributes to the target-binding affinity of Nanosota-3C. We assessed the impact of each individual mutation in Nanosota-3C on its binding to the XBB.1.5 RBD by introducing each of the six mutations separately into Nanosota-3A and examining the binding interaction between each mutant Nanosota-3A and the XBB.1.5 RBD using ELISA (S3 Fig). The data revealed that the A60W mutation significantly increased Nanosota-3A’s binding affinity for the XBB.1.5 RBD, while the other five mutations either slightly increased or did not significantly affect binding affinity. These results align with the structural data, which showed that while the A60W mutation introduced new hydrophobic stacking interactions at the Nanosota-3C/XBB.1.5 interface, the other mutations did not create direct interactions with the XBB.1.5 RBD. Instead, they primarily influenced the conformation of the RBD-binding loop, with their effects appearing to be synergistic.

Next, we examined how the *in vitro* evolved mutations affected the stability of Nanosota-3C. A thermostability assay conducted on Nanosota-3A, -3B, and -3C revealed that, compared to Nanosota-3A, Nanosota-3B showed slightly reduced thermostability, while Nanosota-3C exhibited significantly reduced thermostability (S4 Fig). Nanobodies contain four framework regions that act as structural scaffolds and three complementarity-determining regions (CDRs) responsible for antigen binding [21]. Occasionally, however, frameworks can also directly interact with antigens. In Nanosota-3C, all six mutations are located within the frameworks. It has been shown that mutations in nanobody frameworks and the resulting structural changes can impact nanobody stability [25]. As a result, the significant conformational change in the RBD-binding loop within the framework likely decreased the stability of Nanosota-3C. However, it is important to note that the stability of Nanosota-3C remains within a reasonable range for nanobodies [26]. Additionally, this study serves as a proof of concept that the target-binding affinities of nanobodies can be dramatically increased using a structure-guided *in vitro* evolution approach. Therefore, future applications of this approach involving the *in vitro* evolution of CDR regions are less likely to affect the stability of the nanobodies of interest.

While a specific set of residues in Nanosota-3A was chosen for this study, other residues near the two RBD mutation sites could have also been targeted for *in vitro* evolution. Given the structural flexibility of protein-protein interactions [27,28], there may be multiple ways for a protein to evolve and adapt to mutations in its partner protein. As a result, if different residues in Nanosota-3A had been selected for *in vitro* evolution, the nanobody might have developed a different structural mechanism for binding to the XBB.1.5 RBD with high affinity. Therefore, the residue selections in this study and the resulting structural mechanism for RBD/nanobody binding may not represent the only possible evolutionary pathway for Nanosota-3A to adapt to the XBB.1.5 RBD.

## Discussion

The rapid mutation rate of SARS-CoV-2 necessitates two strategies for antibody treatment: broad-spectrum antibody therapeutics or those that can be quickly modified for new viral variants. Our study explores the second strategy, pioneering a structure-guided *in vitro* evolution of nanobodies to expedite their adaptation to emerging viral variants. From the structural interface between the original nanobody and viral target, we can identify and fully randomize key nanobody residues in close proximity to the viral target’s mutations, and then isolate the nanobody variants that bind most strongly to the mutated viral target. This selection is conducted using a phage display library of nanobody variants encompassing all possible permutations of the randomized residues. The selected nanobody variants are then assessed using a series of *in vitro* and *in vivo* functional assays. Our method is designed to be fast, efficient, and systematic.

Our method surpasses the traditional random *in vitro* evolution techniques in tailoring nanobodies to combat new viral variants. The traditional approach scatters mutations throughout either the entire coding sequence or the CDRs of the nanobody, resulting in a limited and unfocused array of mutant nanobodies within the phage display libraries [21,29–31]. By contrast, our structure-guided *in vitro* evolution method strategically introduces mutations at key residues of the nanobody that are adjacent to the viral target’s mutation sites. This allows for an exhaustive examination of all potential combinations of mutations at these critical sites. Therefore, our structure-guided *in vitro* evolution represents a significant advancement in the field of *in vitro* protein evolution using phage display technology.

Our method has advantages over the immune system in terms of speed and is comparable to the immune system in effectiveness when responding to new viral variant outbreaks. The entire *in vitro* evolution process, from design to selecting the top candidate, can be completed in less than two weeks, which is significantly faster than the rate at which major viral variants emerge [32–34]. In contrast, immunizing animals with antigens from a new virus variant can take several months, not including the additional time required for phage library construction and screening. Moreover, our *in vitro* method can match the natural immune response in generating potent antiviral nanobodies. For instance, Nanosota-3C, developed through our *in vitro* evolution process, demonstrates potency comparable to some of the most effective anti-SARS-CoV-2 nanobodies produced by the immune system [35]. The rapid and efficient production of potent antiviral nanobodies positions structure-guided *in vitro* evolution as a prime strategy for combating emergent viral variants.

Our method confers a distinct advantage to nanobodies in their ability to be rapidly adapted to new viral variants when compared to conventional antibodies. The single-domain structure of nanobodies allows for straightforward cloning into phage vectors and surface display on phages. The selected nanobodies can also be efficiently expressed in bacterial systems for functional characterizations. In contrast, the two-chain structure of conventional antibodies complicates their use in phage display libraries and conventional antibodies also require expression in mammalian cells, creating substantial challenges when attempting to use the same rapid adaptation strategy. Overall, nanobodies are exceptionally well-equipped for this structure-guided *in vitro* evolution approach, standing out as an excellent solution in the face of rapidly evolving viral threats.

In summary, this study has led to the development of a cutting-edge, structure-guided *in vitro* evolution technique that efficiently adapts nanobodies to combat new viral variants. Our proof of concept has successfully demonstrated the technique’s speed and effectiveness. This approach has the potential to broaden the application of nanobodies in antiviral research.

## Methods

### Ethics statement

This study was performed in strict accordance with the recommendations in the Guide for the Care and Use of Laboratory Animals of the National Institutes of Health. All of the animals were handled according to approved institutional animal care and use committee (IACUC) protocols of the University of Iowa (protocol number: 9051795).

### Cell lines, plasmids and viruses

HEK293T cells (American Type Culture Collection) were cultured in Dulbecco’s modified Eagle medium (DMEM) with 10% fetal bovine serum. TG1 *E*. *coli* (Lucigen) and ss320 *E*. *coli* (Lucigen) were grown in 2YT medium. Vero E6 cells (American Type Culture Collection) were cultured in Eagle’s minimal essential medium (EMEM) supplemented with penicillin (100 units/ml), streptomycin (100 μg/ml), and 10% fetal bovine serum. Prototypic SARS-CoV-2 spike gene (GenBank: QHD43416.1) was synthesized (GenScript) with an introduced D614G mutation. Spike genes of Omicron BA.1 subvariant (GISAID: EPI_ISL_6590782.2) and Omicron XBB.1.5 subvariant (GISAID: EPI_ISL_17774216) were derived from the prototypic SARS-CoV-2 spike gene through mutagenesis. Each of the spike genes was cloned into the pcDNA3.1(+) vector.

SARS-CoV-2 spike ectodomains (residues 14–1211, 14–1207, and 14–1207 for prototypic spike, BA.1 spike and XBB.1.5 spike, respectively) and SARS-CoV-2 RBDs (residues 316–526 and 315–525 for BA.1 RBD and XBB.1.5 RBD, respectively) were subcloned into Lenti-CMV vector (Vigene Biosciences) with an N-terminal tissue plasminogen activator (tPA) signal peptide and a C-terminal His tag. Additionally, in these spike ectodomain constructs, the furin motif RRAR was replaced with AGAR and six proline mutations were introduced to the S2 subunit region as previously described [23]. Fc-tagged nanobodies were constructed in the same way as the above RBDs except that a C-terminal human IgG_1_ Fc tag replaced the C-terminal His tag.

Infectious XBB.1.5 was purchased from BEI resources. Experiments involving infectious viruses were conducted at the University of Iowa in approved biosafety level 3 laboratories.

### Structure-guided *in vitro* evolution of Nanosota-3A

To enhance binding to the XBB.1.5 RBD, Nanosota-3A underwent two stages of structure-guided *in vitro* evolution. The first stage involved random mutations at Val50 and Gln58 through PCR, creating a collection of Nanosota-3A mutant genes. These mutations were completely random within the targeted sites on the PCR primers. This collection of Nanosota-3A mutant genes were then cloned into the PADL22c vector and introduced into TG1 cells via electroporation to construct a mutagenic phage display library. Mutant phages with improved binding to XBB.1.5 spike were then selected through bio-panning. After three rounds of bio-panning, the best binding phages were identified, their nanobody genes sequenced, and their nanobodies tested for affinity to XBB.1.5 spike using ELISA. The best target-binding mutant, named Nanosota-3B, contains two mutations, V50F and Q58S. In the second evolution stage, Nanosota-3B was mutated at four additional sites: Met47, Ala60, Asn61, and Phe62, following the same procedure to produce Nanosota-3C, which contains four additional mutations (M47G, A60W, N61L, and F62P).

Mutagenesis primer for Val50 and Gln58 mutations: GAAGCAGCGCGAAATGGTCGCANNNATTAGTAGTATTGCTAGCACGNNNTATGCAAACTTCGTGAAG

Mutagenesis primer for Met47 mutation: GGTTTCGCCAGGCTCTAGGGAAGCAGCGCGAANNNGTCGCATTTATTAGTAGTATTGCTAGC

Mutagenesis primer for Ala60, Asn61 and Phe62 mutations: CATTTATTAGTAGTATTGCTAGCACGAGTTATNNNNNNNNNGTGAAGGGCCGATTCACC

### Protein expression and purification

Nanosota-3A, Nanosota-3B, and Nanosota-3C were expressed and purified as previously described [22]. Briefly, the His-tagged nanobodies were purified from the periplasm of ss320 *E*. *coli* following induction with 1 mM IPTG. The *E*. *coli* cells were harvested and re-suspended in 15 ml TES buffer (0.2 M Tris pH 8, 0.5 mM EDTA, 0.5 M sucrose). The proteins in the supernatant underwent sequential purification using a Ni-NTA column and a Superdex200 gel filtration column (Cytiva).

Prototypic, BA.1, and XBB.1.5 spike ectodomains (all with a C-terminal His tag), BA.1 and XBB.1.5 RBDs (both with a C-terminal His tag), and Nanosota-3A, -3B, and -3C (all with a C-terminal Fc tag) were produced from 293F mammalian cells as previously described [36]. Briefly, lentiviral particles were packaged using plasmids encoding one of the above proteins and then used to infect 293F cells to establish stable cell lines in the presence of Puromycin (Gibco). The proteins were harvested from the supernatants of the cell culture medium, purified using a Ni-NTA column for His-tagged proteins or a Protein A column for Fc-tagged proteins, and further purified on a Superose6 Increase gel filtration column (Cytiva) for spike ectodomains or a Superdex200 gel filtration column (Cytiva) for other proteins.

To prepare the complex of the XBB.1.5 spike ectodomain and Nanosota-3C, the two proteins were incubated at room temperature for 30 minutes and then subjected to Superose6 increase gel filtration column (Cytiva).

### ELISA

ELISA was performed to evaluate the binding interaction between His-tagged SARS-CoV-2 spike ectodomains and either HA-tagged nanobodies (also containing a His tag) or Fc-tagged nanobodies, as previously described [21]. ELISA plates were coated with one of the recombinant SARS-CoV-2 spike ectodomains and then sequentially incubated with nanobodies (either from the ss320 *E*. *coli* supernatant or recombinant nanobodies) and then with either HRP-conjugated anti-HA antibody (1:2,000) (Sigma) for HA-tagged nanobodies or HRP-conjugated anti-Fc antibody (1:3,000) (Jackson ImmunoResearch) for Fc-tagged nanobodies. The ELISA substrate (Invitrogen) was then added, and the reaction was stopped using 1N H_2_SO4. Absorbance at 450 nm was measured using a Synergy LX Multi-Mode Reader (BioTek).

### Surface plasmon resonance

Surface plasmon resonance (SPR) was conducted to measure the binding affinity between nanobodies and SARS-CoV-2 RBDs using a Biacore S200 system (Cytiva), as previously described [21]. Briefly, one of the recombinant RBDs (with a His tag) was immobilized on a CM5 chip (Cytiva) through chemical cross-linking. Nanobodies (with a His tag) were injected at different concentrations from 32 nM to 1200 nM. The resulting data were fitted to a 1:1 binding model using Biacore Evaluation Software (Cytiva).

### Differential scanning fluorimetry (DSF)

To assess the thermostabilities of Nanosota-3A-Fc, -3B-Fc, and -3C-Fc, DSF experiments were performed. Each sample, consisting of 0.02 mg of individual nanobody, was mixed with 1.25 μl of diluted Protein Thermal Shift Dye (Thermo Fisher) in 10 μl of PBS (pH 7.4). The mixture was prepared in a 96-well qPCR plate. Protein stability measurements, indicated by the fluorescence signal during protein denaturation, were conducted in a PCR instrument (Applied Biosystems) using a temperature ramp from 25°C to 99°C at a rate of 0.05°C/s. Data collection was performed using real-time PCR software. The negative first derivative of the fluorescence signal was plotted against temperature, with the peak indicating the melting temperature (T_m_).

### Pseudovirus entry assay

SARS-CoV-2 pseudovirus entry assay was carried out to measure the neutralizing potencies of nanobodies against SARS-CoV-2 pseudoviruses, as previously described [36]. Briefly, pseudoviruses were prepared by co-transfecting HEK293T cells with a pcDNA3.1(+) plasmid encoding the spike protein from one of the SARS-CoV-2 variants, a helper plasmid psPAX2, and a reporter plasmid plenti-CMV-luc. After 72 hours, pseudoviruses were collected, incubated with nanobodies at varying concentrations at 37°C for 1 hour, and then used to enter HEK293T cells expressing human ACE2. Following an additional 60 hours, cells were lysed. Portions of the cell lysates were transferred to new plates, a luciferase substrate was introduced, and Relative Light Units (RLUs) were measured using an EnSpire plate reader (PerkinElmer). The efficacy of each nanobody was determined and expressed as the concentration capable of inhibiting pseudovirus entry by 50% (IC_50_).

### Evaluation of nanobody potency in mouse model

The efficacy of Nanosota-3C-Fc against infectious XBB.1.5 *in vivo* was evaluated through a SARS-CoV-2 challenge experiment in a mouse model, as previously described [22]. Briefly, C57BL/6 mice (3–4 month old) were randomly separated into 3 groups. All mice were challenged via intranasal inoculation of infectious XBB.1.5 (1 x 10^4^ PFU/mouse) in a volume of 50 μl DMEM. In the pre-treatment group (n = 5), mice received Nanosota-3C-Fc (10 mg/kg weight) via intraperitoneal delivery at 24 hours pre-challenge. In the post-treatment group (n = 5), mice received Nanosota-3C-Fc (10 mg/kg weight) via intraperitoneal delivery at 4 hours post-challenge. In the control group (n = 5), mice were administered PBS buffer at 4 hours post-challenge. The virus titers in the lungs of the mice were measured using a virus plaque assay, as previously described [22]. Briefly, mice were euthanized on day 2 post-challenge and lung tissue homogenate supernatants were collected. 12-well plates of Vero E6 cells overexpressing ACE2 and TMPRSS2 were inoculated with serially diluted lung homogenates (in DMEM) and then incubated at 37°C in 5% CO_2_ for 1 hour with gently shaking every 15 minutes. Then the inocula were removed and the plates were overlaid with 0.6% agarose containing 2% FBS. After 3 days, the overlays were removed, and the plaques were visualized via staining with 0.1% crystal violet. Virus titers were quantified as PFU per ml tissue.

### Cryo-EM grid preparation and data acquisition

Cryo-EM data collection was conducted as previously described [22]. Briefly, 4 μl complex (at 2 μM) of the XBB.1.5 spike ectodomain and Nanosota-3C was applied to freshly glow-discharged Quantifoil R1.2/1.3 300-mesh copper grids (EM Sciences) and blotted for 4 seconds at 22°C under 100% chamber humidity and plunge-frozen in liquid ethane using a Vitrobot Mark IV (FEI). Cryo-EM data were collected using EPU (Thermo Fisher) equipped with a K3 direct electron detector and with a Biocontinuum energy filter (Gatan) at a nominal magnification of 130,000x (corresponding to 0.664 Å per pixel) (S1 Table).

### Image processing

Image processing was performed as previously described [22]. Briefly, cryo-EM data were processed using cryoSPARC v3.3.2 [37]. Dose-fractionated movies were subjected to Patch motion correction with MotionCor2 [38] and Patch CTF estimation with CTFFIND-4.1.13 [39]. Particles were then picked using Blob picker and Template picker in cryoSPARC v3.3.2 and subjected to the Remove Duplicate Particles Tool. Junk particles were removed through three rounds of 2D classifications. Particles from the good 2D classes were used for Ab-initio Reconstruction of four maps. The initial models were set as the starting references for heterogeneous refinement (3D classification). The selected 3D classes were then subjected to further non-uniform and CTF refinements, generating the final maps. To improve densities of the RBD and nanobody interface, particles in the good 3D class were imported into RELION-4.0 [40] using the csparc2star.py module (UCSF pyem v0.5. Zenodo) and subjected to signal subtraction to keep only the receptor-binding subunit of the spike and the nanobody in RELION-4.0. This was then followed by local refinements in cryoSPARC v3.3.2. Resolutions of the maps were determined by gold-standard Fourier shell correlation (FSC) at 0.143 between the two half-maps. Local resolution variations were estimated from the two half-maps in cryoSPARC v3.3.2 (S2 and S4 Figs).

### Cryo-EM model building and refinement.

Cryo-EM model building and refinement were carried out as previously described [22]. Briefly, initial model building of the spike/nanobody complex was performed in Coot-0.8.9 [41] using PDB 8VKL as the starting model. Previously published structure of Nanosota-3A was used as the initial model for Nanosota-3C [22]. Several rounds of refinement in Phenix-1.16 [42] and manually building in Coot-0.8.9 were performed until the final reliable model was obtained. In the model, standing-up RBDs and their bound nanobodies are generally flexible and hence they were fitted into the density as rigid bodies. In the local map of the XBB.1.5 spike/Nanosota-3C complex, an atomic model was built at the interface between the lying-down RBD and Nanosota-3C (S1 Table). Figures were generated using UCSF Chimera X v0.93 [43] and PyMol v2.5.2 [44].

## Supporting information

S1 Fig Flow chart of cryo-EM image processing and 3D reconstruction for the XBB.1.5 spike/Nanosota-3C complex. Representative raw cryo-EM images and 2D classes are shown. 3D refinements using all the particles from the good 3D classes produced a 3.19 Å map. Further local refinement improved the density for the bound nanobody. The angular distribution plot, final maps, half-map FSC curves, and accompanying local resolution illustrations are enclosed in the dashed black boxes.
(TIF)

S2 Fig Cryo-EM densities of the XBB.1.5 spike/Nanosota-3C interface. The XBB.1.5 chain is shown in magenta, and the Nanosota-3C chain is shown in blue. All residues are represented as gray sticks.
(TIF)

S3 Fig Contribution of individual mutations in Nanosota-3C to the protein’s target-binding affinity. **(A)** The binding interactions between the XBB.1.5 spike ectodomain and Nanosota-3A containing one of the six mutations evolved in Nanosota-3C were evaluated using ELISA at different nanobody concentrations. Nanosota-3A and -3C were used as controls. Error bars represent SEM (n = 3). **(B)** ELISA data at the highest concentration of the nanobodies. A Student’s two-tailed t-test was performed to analyze the statistical differences between Nanosota-3A and each of the other nanobodies; the results are indicated above each bar. *** *p*< 0.001. * *p*< 0.05. n.s. not statistically significant.
(TIF)

S4 Fig Thermal stabilities of Nanosota-3A-Fc, -3B-Fc, and -3C-Fc. The thermal stabilities of the three nanobody variants were measured using a differential scanning fluorimetry (DSF) assay. Protein stability was assessed by monitoring the fluorescence signal during protein denaturation at increasing temperatures. The negative first derivative of the fluorescence signal was plotted against temperature, with the peak indicating the melting temperature (T_m_). RFU: relative fluorescence units.
(TIF)

S1 Table Cryo-EM data collection, refinement and validation statistics of the XBB.1.5 spike/Nanosota-3C complex. (TIF)
