# Supplementary figures and images for: Structure-guided in vitro evolution of nanobodies targeting new viral variants
Source: PLoS Pathog. 2024 Sep 26;20(9):e1012600. doi: 10.1371/journal.ppat.1012600 (PMC11460708; doi:10.1371/journal.ppat.1012600)

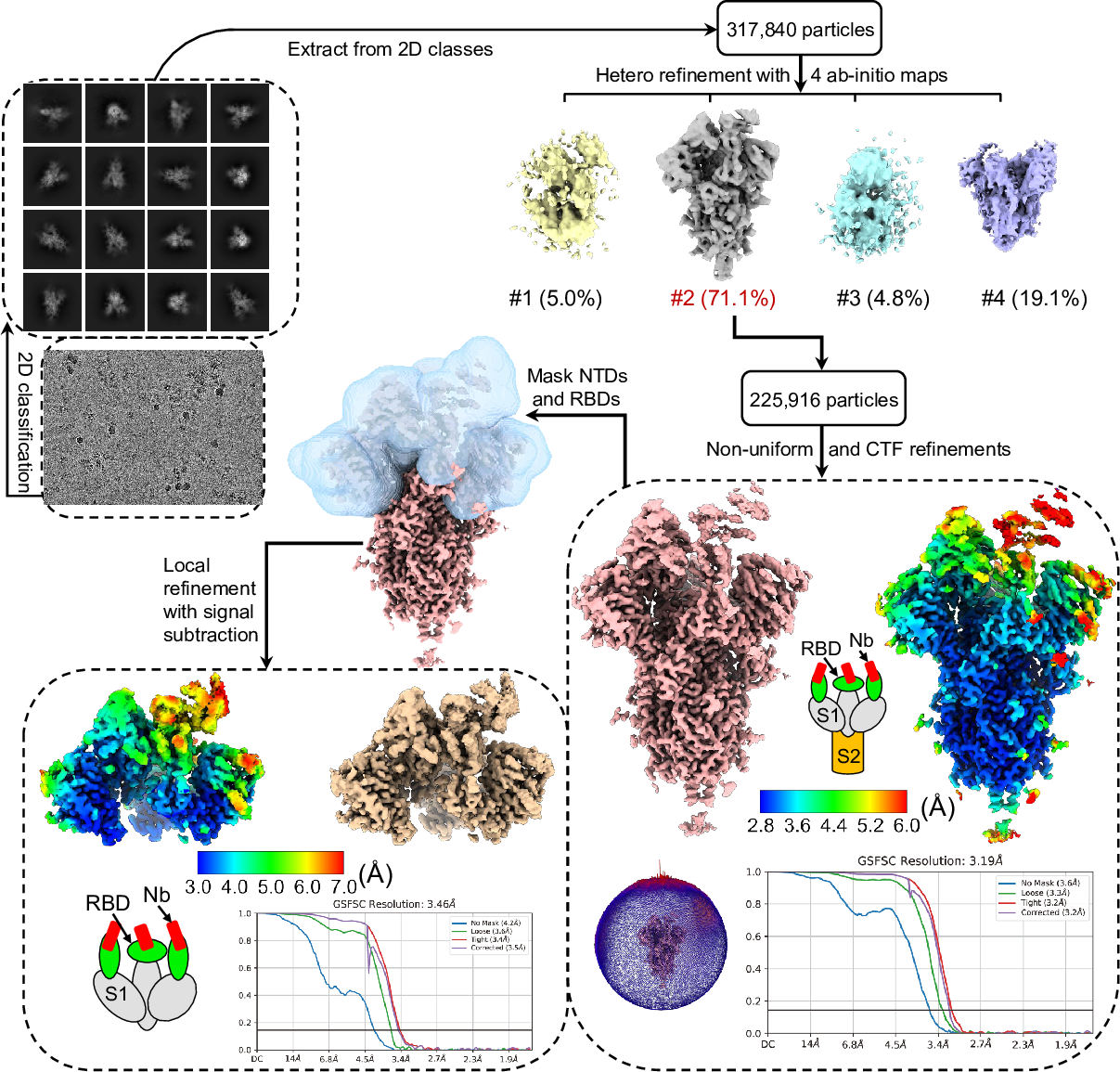

Supplement: S1 Fig — Representative raw cryo-EM images and 2D classes are shown. 3D refinements using all the particles from the good 3D classes produced a 3.19 Å map. Further local refinement improved the density for the bound nanobody. The angular distribution plot, final maps, half-map FSC curves, and accompanying local resolution illustrations are enclosed in the dashed black boxes. (TIF) [file ppat.1012600.s001.tif]

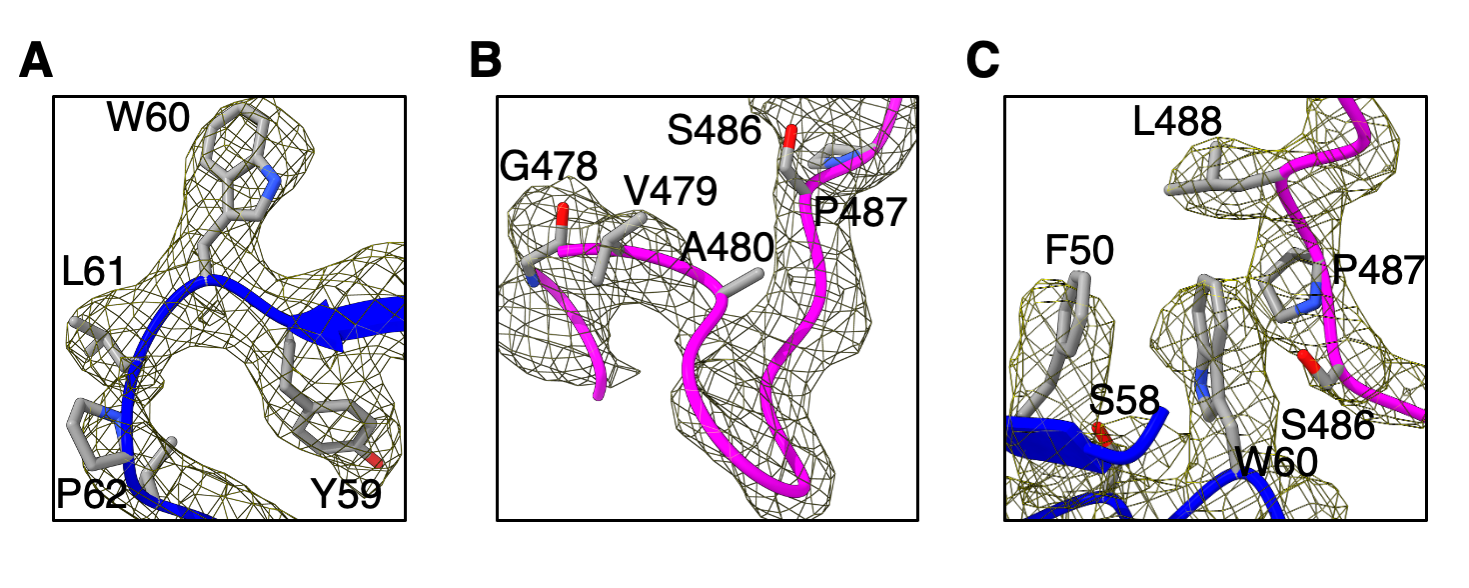

Supplement: S2 Fig — The XBB.1.5 chain is shown in magenta, and the Nanosota-3C chain is shown in blue. All residues are represented as gray sticks. (TIF) [file ppat.1012600.s002.tif]

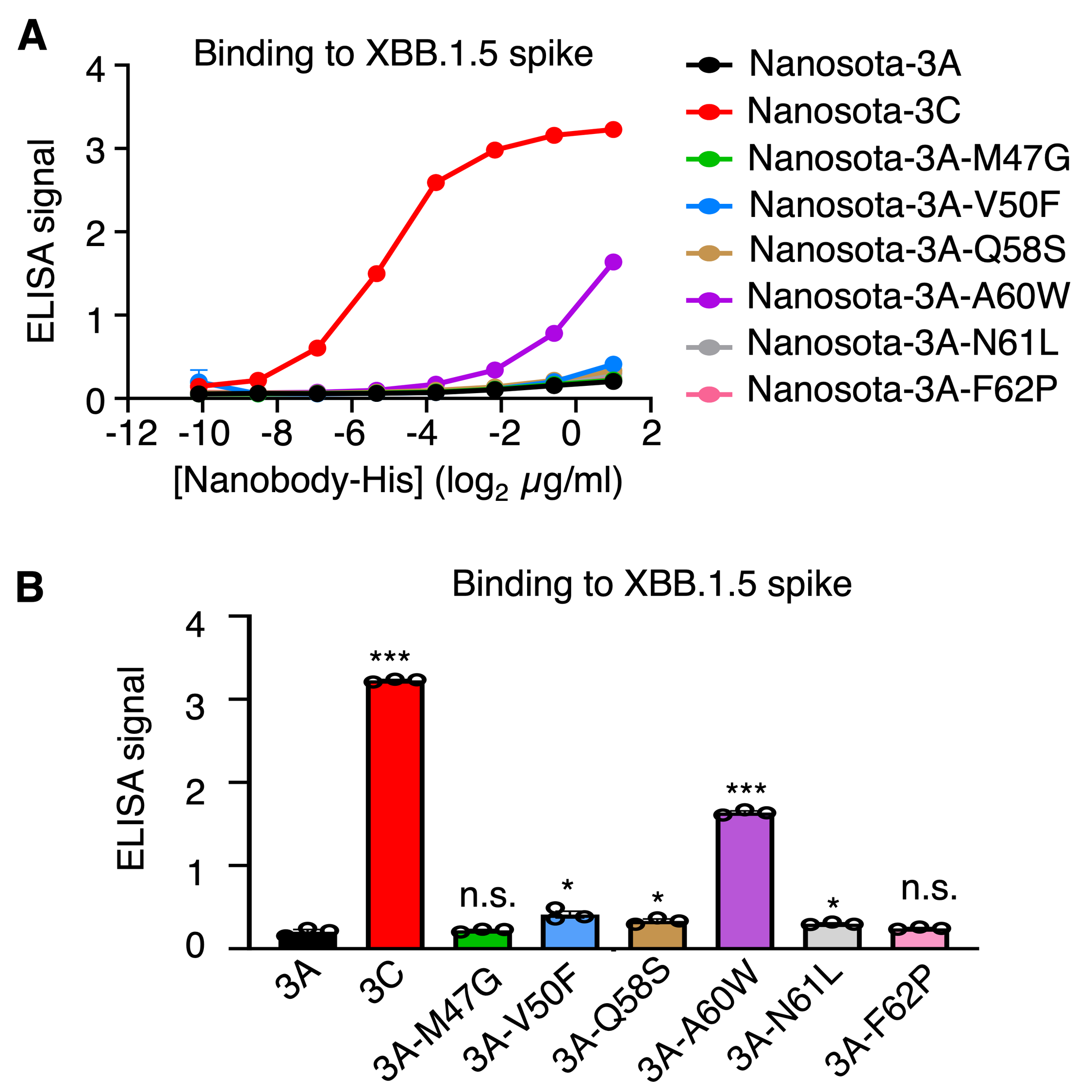

Supplement: S3 Fig — (A) The binding interactions between the XBB.1.5 spike ectodomain and Nanosota-3A containing one of the six mutations evolved in Nanosota-3C were evaluated using ELISA at different nanobody concentrations. Nanosota-3A and -3C were used as controls. Error bars represent SEM (n = 3). (B) ELISA data at the highest concentration of the nanobodies. A Student’s two-tailed t-test was performed to analyze the statistical differences between Nanosota-3A and each of the other nanobodies; the results are indicated above each bar. *** p< 0.001. * p< 0.05. n.s. not statistically significant. (TIF) [file ppat.1012600.s003.tif]

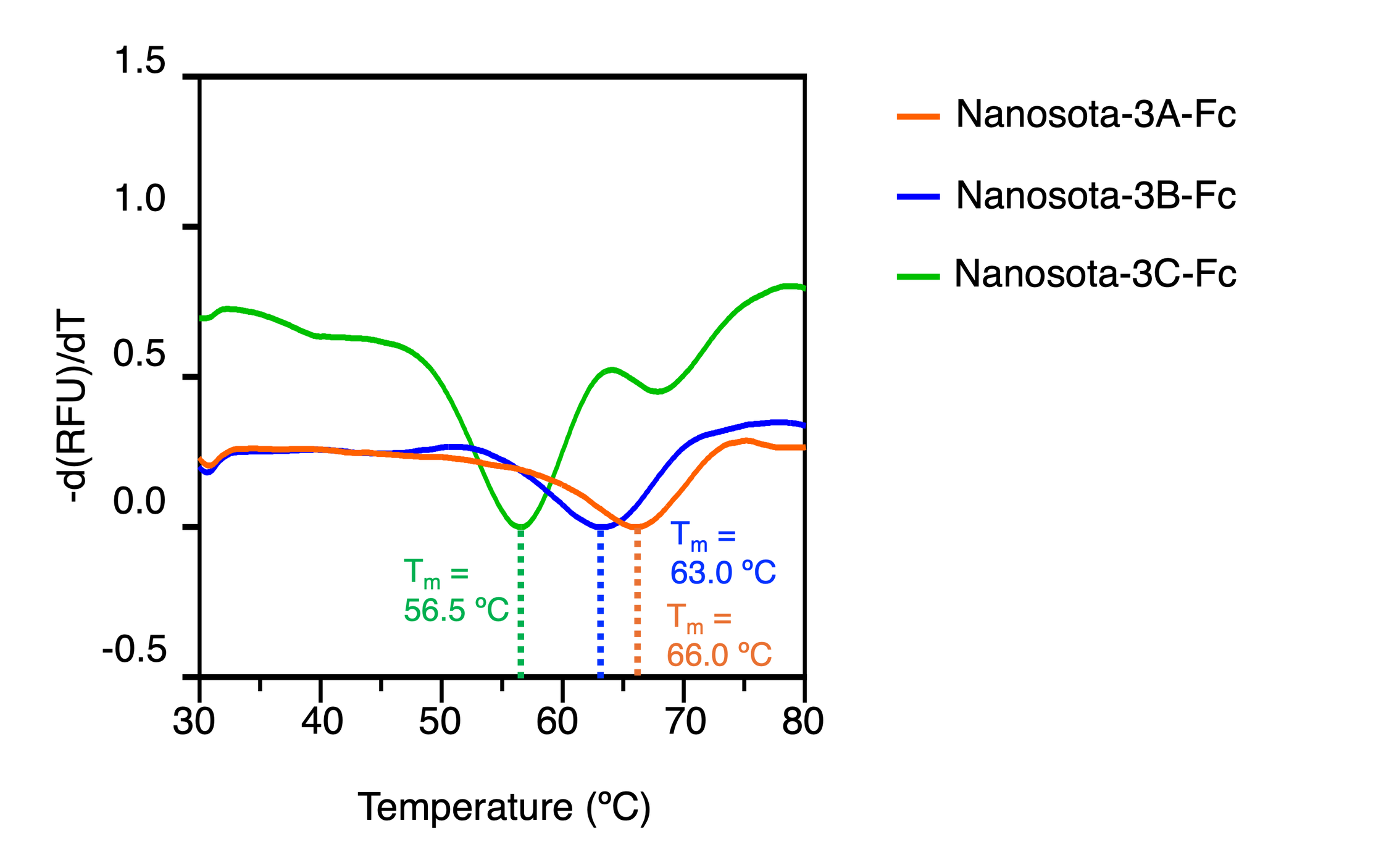

Supplement: S4 Fig — The thermal stabilities of the three nanobody variants were measured using a differential scanning fluorimetry (DSF) assay. Protein stability was assessed by monitoring the fluorescence signal during protein denaturation at increasing temperatures. The negative first derivative of the fluorescence signal was plotted against temperature, with the peak indicating the melting temperature (Tm). RFU: relative fluorescence units. (TIF) [file ppat.1012600.s004.tif]

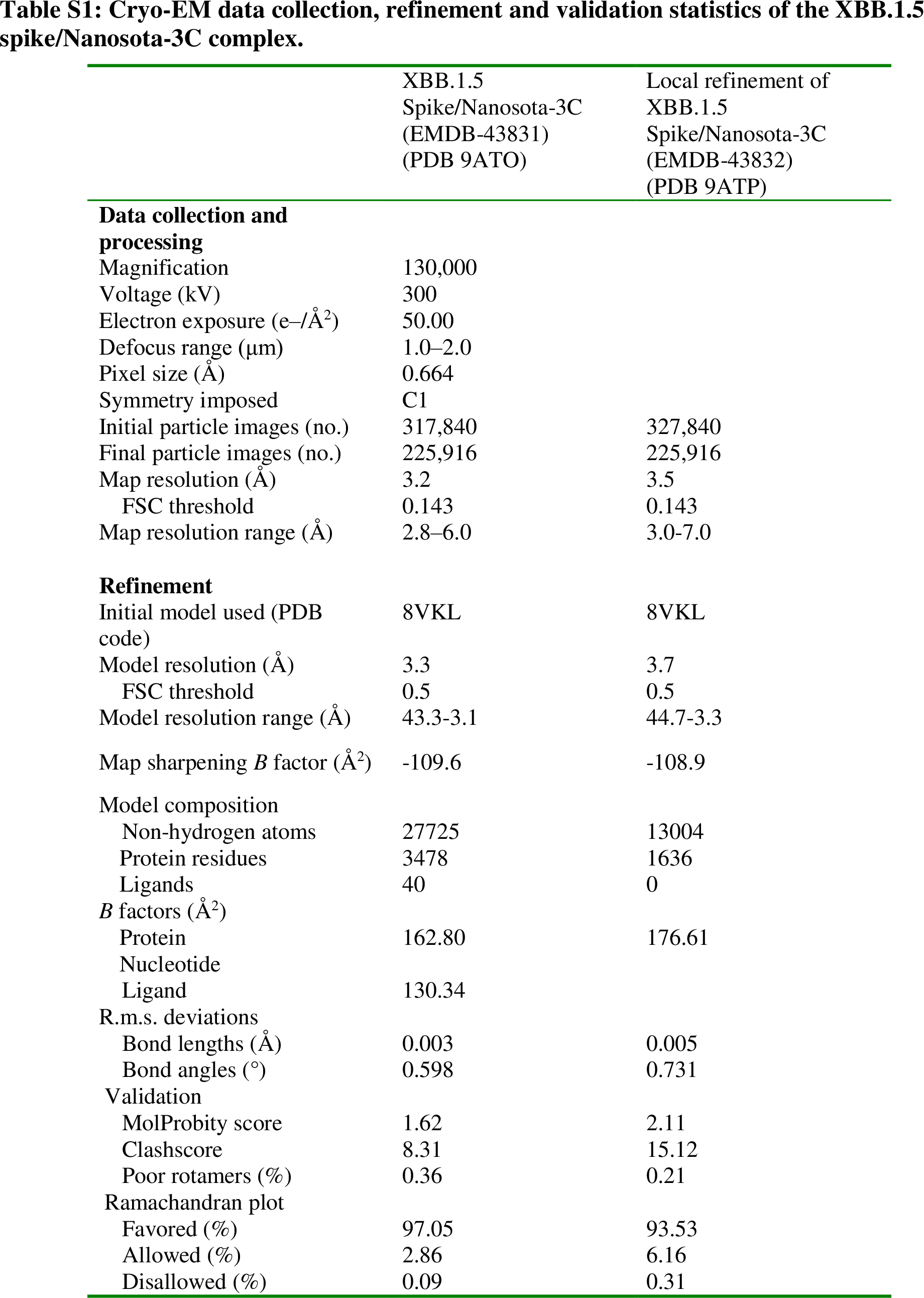

Supplement: S1 Table — (TIF) [file ppat.1012600.s005.tif]
